# XIAOPI formula inhibits the pre-metastatic niche formation in breast cancer via suppressing TAMs/CXCL1 signaling

**DOI:** 10.1186/s12964-020-0520-6

**Published:** 2020-03-26

**Authors:** Yifeng Zheng, Neng Wang, Shengqi Wang, Bowen Yang, Honglin Situ, Lidan Zhong, Yi Lin, Zhiyu Wang

**Affiliations:** 1 Integrative Research Laboratory of Breast Cancer, the Research Centre of Integrative Medicine, Discipline of Integrated Chinese and Western Medicine & The Second Affiliated Hospital of Guangzhou University of Chinese Medicine, Guangzhou, 510006 Guangdong China; 2 Guangdong Provincial Key Laboratory of Clinical Research on Traditional Chinese Medicine Syndrome, Guangdong Provincial Academy of Chinese Medical Sciences, Guangdong Provincial Hospital of Chinese Medicine, Guangzhou, 510006 Guangdong China; 3 College of Basic Medicine, Guangzhou University of Chinese Medicine, Guangzhou, 510006 Guangdong China; 4 School of Chinese Medicine, Hong Kong Baptist University, Hong Kong, Special Administrative Region China

**Keywords:** XIAOPI formula, Premetastatic niche, Tumor-associated macrophages, CXCL1, Breast Cancer

## Abstract

**Background:**

Recent findings suggested that premetastatic niche (PMN) is a prerequisite in mediating cancer metastasis. Previously we demonstrated that XIAOPI formula could inhibit breast cancer lung metastasis via inhibiting tumor associated macrophages (TAMs)-secreted CXCL1. Herein, we aimed to explore the effects of XIAOPI formula on preventing breast cancer PMN formation and its underlying molecular mechanisms.

**Methods:**

CXCL1 expression of TAMs was detected by qPCR and Western blotting assay. The influences of XIAOPI formula on the proliferation of TAMs and 4 T1 in the co-culture system were tested by CCK8 or EdU staining. Transwell experiment was applied to determine the effects of XIAOPI formula on the invasion ability of HSPCs and 4 T1. Breast cancer xenografts were built by inoculating 4 T1 cells into the mammary pads of Balb/c mice and lung metastasis was monitored by luciferase imaging. Immune fluorescence assay was used to test the epithelial-mesenchymal transition process and PMN formation in the lung tissues. The effects of XIAOPI formula on TAMs phenotype, hematopoietic stem/progenitor cells (HSPCs) and myeloid-derived suppressor cells (MDSCs) were determined by flow cytometry.

**Results:**

It was found that XIAOPI formula could inhibit the proliferation and polarization of M2 phenotype macrophages, and reduce CXCL1 expression in a dose-dependent manner. However, M1 phenotype macrophages were not significantly affected by XIAOPI formula. TAMs/CXCL1 signaling was subsequently found to stimulate the recruitment of c-Kit^+^/Sca-1^+^ HSPCs and their differentiation into CD11b^+^/Gr-1^+^ MDSCs, which were symbolic events accounting for PMN formation. Moreover, XIAOPI formula was effective in inhibiting HSPCs activation and suppressing the proliferation and metastasis of breast cancer cells 4 T1 induced by HSPCs and TAMs co-culture system, implying that XIAOPI was effective in preventing PMN formation in vitro. Breast cancer xenograft experiments further demonstrated that XIAOPI formula could inhibit breast cancer PMN formation and subsequent lung metastasis in vivo. The populations of HSPCs in the bone marrow and MDSCs in the lung tissues were all remarkably declined by XIAOPI formula treatment. However, the inhibitory effects of XIAOPI formula could be relieved by CXCL1 overexpression in the TAMs.

**Conclusions:**

Taken together, our study provided preclinical evidence supporting the application of XIAOPI formula in preventing breast cancer PMN formation, and highlighted TAMs/CXCL1 as a potential therapeutic strategy for PMN targeting therapy.

**Video Abstract**

**Graphical abstract:**

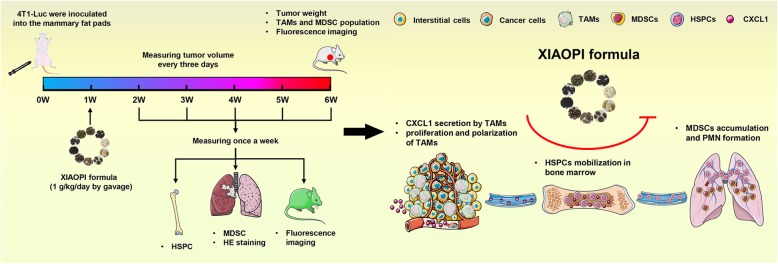

## Background

Breast cancer is the most common malignancy among women worldwide with the average morbidity of 21.6/10000 [1]. Although great advances have been made in breast cancer diagnosis and treatment, distant metastasis is always a life-threaten event influencing clinical prognosis. It is reported that 6% patients were diagnosed with metastatic breast cancer at first visit, and 20–50% patients with primary breast cancer experienced distant metastases after 3–10 years [2]. Therefore, elucidation of molecular mechanisms underlying breast cancer metastasis and the development of targeted strategies has become an urgent issue facing by oncologists globally.

Since the “seed and soil” theory initially proposed by Paget et al. in 1889, increasing studies have demonstrated the close correlation between the primary tumor and the microenvironment of premetastatic sites. In 2005, a groundbreaking research done by Kaplan et al. proved that factors secreted by primary tumor cells shaped the premetastatic niche (PMN) by recruiting bone marrow-derived VEGFR1^+^ progenitor cells [3]. Since then, much attention has been paid to explore the critical roles of non-malignant cells and factors in facilitating PMN formation. For instance, hypoxia condition was found to promote bone marrow-derived cells (BMDCs) recruitment in the lung tissue and finally compromised NK cell cytotoxicity in PMN [4]. Also, Jagged2 was unregulated in the bone marrow under hypoxia, and subsequently enhancing the self-renewal ability of cancer stem-like cells by activating Notch signaling [5]. Besides, noncoding RNAs act as tumor-derived molecular components in inducing PMN formation. Breast cancer-secreted miR-122 was reported to inhibit glucose uptake by niche cells and finally supported PMN establishment [6]. Recent findings also suggested that microvesicles and exosomes released from cancer cells favor PMN formation in a variety of malignancies [7]. Melanoma-secreted exosomes were found to educate bone marrow progenitor cells toward a pro-metastatic phenotype through the mesenchymal-epithelial transition [8]. What’s more important, stromal cells were highly implicated in mediating PMN formation [9]. Neutrophils were reported to facilitate ovarian cancer PMN formation in the omentum [10]. Tumor associated macrophages (TAMs)-secreted CXCL1 was demonstrated to recruit CXCR2^+^ myeloid suppressor cells (MDSCs) to promote liver PMN formation in a colorectal cancer xenograft [11]. Taken together, PMN theory has shifted our attention from cancer cell killing strategy to niche component regulation in the perspective of metastasis prevention.

It is noteworthy that PMN process could be divided into 3 phases: (1) tumor-secreted soluble cytokines or components were released into the circulation; (2) activation and directional recruitment of immune suppressor cells in the bone marrow; (3) cellular and matrix remodeling in the PMN [12]. Therefore, PMN formation is a holistic process which involves multiple organs, various cells, and numerous targets. Coincidently, Traditional Chinese Medicine (TCM) is also appreciated for disease treatment and prevention by its holistic opinion. In the past few years, numerous studies demonstrated that TCM herbs or formulas could not only inhibit cancer growth or metastasis via killing cancer cells, but also improve the immune suppression microenvironment in the tumor or distant organs. Some studies have demonstrated that TCM formulas were capable of preventing metastasis by enhancing the immune function of T cells, activating mononuclear macrophages and NK cells, improving antigen presentation capabilities of dendritic cells and inhibiting tumor immune escape, etc. [13–15]. Our previous study has found that XIAOPI formula could suppress the pulmonary metastasis of breast cancer. Network pharmacology analysis combined with experimental validation discovered that XIAOPI formula plays the anti-cancer effects mainly via inhibiting the TAMs/CXCL1 pathway [16]. However, the effects of XIAOPI formula on preventing PMN formation and its underlying mechanisms still remained unknown.

In this study, we found that XIAOPI formula could inhibit TAMs/CXCL1 signaling, subsequently decline HSPCs activation and differentiation into MDSCs, and finally inhibit breast cancer cell proliferation and metastasis. In vivo studies also demonstrated that XIAOPI formula could inhibit breast cancer growth and metastasis. More importantly, XIAOPI formula was demonstrated to inhibit PMN formation by suppressing HSPCs recruitment and MDSCs accumulation in the lung tissues. Our study not only provided evidence supporting the application of XIAOPI formula in preventing breast cancer PMN formation, but also highlighted the critical role of TAMs/CXCL1 as a potential therapeutic target in preventing breast cancer metastasis.

## Methods

### Cell culture and induction

The mouse breast cancer cell line 4 T1 and mouse macrophage cell line Raw264.7 were obtained from the American Type Culture Collection. 4 T1 and Raw264.7 cells were cultured in DMEM and RPMI 1640 medium respectively, supplemented with 10% fetal bovine serum. All the cells were maintained at 37 °C in a humidified incubator containing 5% CO_2_. 100 ng/ml lipopolysaccharide and 100 ng/ml IFN-γ (PeproTech China, Suzhou, Jiangsu) were used to induce RAW264.7 into M1 phenotype. 40 ng/ml IL-4 and 40 ng/ml IL-13 (PeproTech China, Suzhou, Jiangsu) were used to induce the transformation of RAW264.7 macrophages into M2 phenotype TAMs.

### Preparation and quality control of XIAOPI formula

XIAOPI formula was extracted from a mixture of 10 herbs including epimedium brevicornum, *Cistanche deserticola*, leonurus heterophyllus, salvia miltiorrhiza, *Curcuma aromatica*, rhizoma curcumae, *Ligustrum lucidum*, radix polygoni multiflori preparata, *Crassostrea gigas* and carapax trionycis by refluxing extraction method. Its quality control was applied by detecting the high performance liquid chromatography fingerprints between different batches. The detailed preparation and quality control method has been previously reported [16].

### HSPCs preparation from mouse bone marrow

Murine bone marrow cells in the femur and tibia were flushed out using a syringe under sterile conditions. Subsequently, the Lineage^−^ and c-Kit^+^ cells were isolated using MACS separators according to the manufacturer’s protocol of Lineage Cell Depletion Kit (130–090-858, Miltenyi Biotec China, Guangzhou, China) and CD117 MicroBeads (130–091-224, Miltenyi Biotec China, Guangzhou, China). Cells sorted by MACS were further incubated with c-Kit (12–1171-81, Thermo Fisher Scientific, Shanghai, China) and Sca-1 (11–5981-81, Thermo Fisher Scientific, Shanghai, China) antibodies, and finally isolated by flow cytometry sorting. HSPC cells were identified as the cell population of c-Kit^+^ and Sca-1^+^.

### Western blotting

Cells were treated as indicated and then lysed by RIPA (Beyotime Biotechnology, Shanghai, China). Protein concentration was quantified with the Bicinchoninic Acid Kit (Sigma-Aldrich, Shanghai, China) according to the manufacturer’s instructions. Equal amounts of protein (50 μg) were loaded for SDS-PAGE electrophoresis, transferred to a polyvinylidene fluoride microporous membrane (Millipore, Billerica, MA). The signals were probed with primary antibodies and amplified by the secondary antibodies. The primary antibodies included ARG1 (DF6657, Affinity Biosciences, Cincinnati, OH, USA), iNOS (18985–1-AP, Proteintech, Rosemont, IL, USA), CXCL1(AF5403, Affinity Biosciences, Cincinnati, OH, USA), CXCR2 (20634–1-AP, Proteintech, Rosemont, IL, USA), β-actin antibody (4970, Cell Signaling Technology, Danvers, MA, USA), MMP2 (A6247, ABclonal Technology Cambridge, Boston, USA), MMP9 (10375–2-AP, Proteintech, Rosemont, IL, USA). Finally, the bands were imaged through the ECL Advance reagent (Tanon Science & Technology, Shanghai, China).

### Flow cytometry assay

Cells were harvested, washed, and resuspended in 100 μl PBS solution at a density of 1 × 10^6^ cells. For detection of M2 polarization, FITC-conjugated F4/80 antibody (SC-71085, Santa Cruz Biotechnology, Santa Cruz, CA, USA), PE-conjugated CD206 antibody (141,705, Biolegend, San Diego, CA, USA), PE-conjugated CD206 antibody (17–4801-80, Thermo Fisher Scientific, Hudson, USA), APC-conjugated CD86 antibody (558,703, BD Biosciences, San Jose, CA, USA), ARG1 Antibody (DF6657, Affinity Biosciences, Cincinnati, OH, USA) were used. Alexa Fluor488 probe was used to bind with Arg1 antibody. For MDSCs analysis, Alexa Fluor® 488 Gr-1 antibody (108,419, Biolegend, San Diego, CA, USA) and PE anti-mouse/human CD11b Antibody (101,207, Biolegend, San Diego, CA, USA) were used. For identification of TAMs in mouse breast tumors and lung lesions, cells were incubated with CD45-PE-Cy7 (25–0451-82, Thermo Fisher Scientific, Hudson, USA), FITC-conjugated F4/80 antibody (SC-71085, Santa Cruz Biotechnology, Santa Cruz, CA, USA) and PE-conjugated CD206 antibody (141,705, Biolegend, San Diego, CA, USA). The HSPCs were identified by analyzing surface markers of FITC-conjugated Sca-1 (11–5981-81, Thermo Fisher Scientific, Hudson, USA) and PE-conjugated c-Kit (12–1171-81, Thermo Fisher Scientific, Hudson, USA) using flow cytometer. After incubation, cells were washed with PBS and subjected into the FACSAria III flow cytometer (BD Biosciences, San Jose, CA, USA).

### Cell viability

CCK-8 assay was applied to assess the cell viability. Induced M1 and M2 phenotype macrophage were seeded at a density of 3 × 10^3^ cells into the 96-well plate. After attachment, cells were treated with serial concentration gradients of XIAOPI formula for 24, 48 and 72 h. The cell viability was measured by CCK-8 reagent (Beyotime Biotechnology, Shanghai, China) according to the manufacturer’s instructions. Three independent repetitive experiments were conducted.

### RT-qPCR analysis

Total RNA was extracted with RNAiso Plus Reagent (Takara BIO, Japan) and reverse transcribed to complementary cDNA using the PrimeScript™ RT reagent Kit with gDNA eraser (Takara BIO, Japan) following the manufacturer’s instructions. The RT-PCR was performed on the Applied Biosystems ViiA7 Real-Time PCR System (Thermo Fisher Scientific, Hudson, USA) using the SYBR® Premix Ex TaqTM II kit (Takara BIO, Japan) following the manufacturer’s instruction. Primer sequences of mouse *β-actin* were 5′-GGAGGGGGTTGAGGTGTT-3′ (forward) and 5′-GTGTGCACTTTTATTGGTCTCAA-3′ (reverse). Primer sequences of mouse *CXCL1* were 5′-GACTCCAGCCACACTCCAAC-3′ (forward) and 5′-TGACAGCGCAGCTCATTG-3′ (reverse). The relative mRNA levels were compared using the 2^-ΔΔCt^ method.

### Transwell invasion assay

To investigate the trending mobility of HSPCs under different interventions, the transwell invasion assay was performed. Briefly, HSPCs were seeded in the upper transwell chamber at a density of 2 × 10^5^ cells per well. Then, HSPCs were treated by 500 μg/ml XIAOPI formula, conditional medium (CM) of TAMs, or 20 ng/ml CXCL1 respectively. After 24 h incubation, the cells that penetrated the filter were fixed with 4% paraformaldehyde, followed by 0.1% Coomassie blue staining. Meanwhile, HSPCs entering the lower chamber were also counted. For co-culture of 4 T1 and HSPCs, the 24-well transwell co-culture system was used. In brief, 4 T1 at a density of 5 × 10^4^ cells and were placed in the upper transwell chamber. While HSPC at a density of 2 × 10^5^ cells were seeded in the lower transwell chamber. Transwell inserts were separated by permeable membrane which allowed the free exchange of media and soluble molecules. Then, CM of TAMs, 500 μg/ml XIAOPI formula, 20 ng/ml CXCL1 or 50 ng/ml neutralizing antibody of CXCL1 were added into 4 T1 cells as arranged in advance. Similarly, after 24 h incubation, the 4 T1 cells that penetrated the filter were stained by 0.1% Coomassie blue.

### EdU assay

4 T1 cells in logarithmic growth phase were seeding into 35 mm laser confocal culture dish at a density of 5 × 10^4^ per well. Then, the cells were treated with control medium, 500 μg/ml XIAOPI formula, CM of TAMs, 20 ng/ml CXCL1 or 50 ng/ml neutralizing antibody of CXCL1 as arranged in advance. After treatment for 24 h, each well was replaced with medium containing 50 μM EdU reagent and incubated for 2 h. Then the cells were washed three times with PBS and fixed with 4% formaldehyde for 30 mins at room temperature. Thereafter, 0.5% TritonX-100 was used for cell permeabilization. Finally, the cells were stained with Apollo® in dark for 30 mins at room temperature, rinsed with PBS and observed by a laser scanning confocal microscope.

### Immunofluorescence analysis

For cell immunofluorescence, cells were plated at the 35 mm laser confocal dishes. After treatment, the cells were fixed with 4% paraformaldehyde for 20 mins, washed three times with PBS, permeabilized with 0.25% Triton X-100 for 20 mins and then blocked in 5% BSA for 30 mins at room temperature. For EMT detection, treated cells were incubated with E-cadherin antibody (20874–1-AP, Proteintech, Rosemont, IL, USA) and vimentin antibody (10366–1-AP, Proteintech, Rosemont, IL, USA) overnight at 4 °C, followed by an incubation with the Alexa Fluor® 488 conjugated-anti-rabbit IgG (4412, Cell Signaling Technology, Danvers, MA, USA) and the Alexa Fluor® 555 conjugated-anti-rat IgG (4417, Cell Signaling Technology, Danvers, MA, USA) for 2 h. For tissue immunofluorescence, the frozen tissue sections were pretreated as same as above, and were incubated the Alexa Fluor® 488 Gr-1 antibody (108,419, Biolegend, San Diego, CA, USA) and Alexa Fluor® 594 CD11b antibody (101,207, Biolegend, San Diego, CA, USA) overnight at 4 °C. In addition, CK-19 antibody (10712–1-AP, Proteintech, Rosemont, IL, USA) was also incubated simultaneously, followed by coupling with the fluorescent antibody of Alexa Fluor® 647 conjugated-anti-rabbit IgG (4414, Cell Signaling Technology, Danvers, MA, USA). The nucleus was stained by DAPI (Sigma-Aldrich, Shanghai, China) for 20 mins at room temperature. In the end, the fluorescence was visualized by the LMS710 confocal microscope (ZEISS, Jena, Germany).

### Animal experiments

Five-week-old female Balb/c mice were obtained from the Beijing Vital River Laboratory Animal Technology Co., Ltd. All in vivo experiments were reviewed and approved by the Institutional Animal Care and Use Committee of Guangdong Provincial Hospital of Chinese Medicine. The mice were raised in the Experimental Animal Center of Guangdong Provincial Hospital of Chinese medicine under specific pathogen-free conditions with the ambient temperature of 20–25 °C and 45–50% relative humidity, and given sterilized food and water. The rearing facility was maintained on a 12 h light-dark cycle. To established the lung metastasis of breast cancer in mice, luciferase gene-tagged 4 T1 (4T1-Luc) were inoculated subcutaneously into the mammary fat pads of mice at a density of 2 × 10^6^. The mice were randomized into 6 groups (*n* = 12), including saline (gavage), XIAOPI formula (1 g/kg/day by gavage), TAMs (co-injection of 4 T1-Luc cells with M2 phenotype RAW264.7 at the ratio of 1:3), TAMs + XIAOPI formula (4 T1-Luc and M2 phenotype RAW264.7 co-injection, followed by XIAOPI formula treatment), shCXCL1/TAMs (co-injection of 4 T1-Luc and M2 phenotype RAW264.7 with CXCL1 knockdown), rCXCL1/TAMs + XIAOPI formula (co-injection of 4 T1-Luc and M2 phenotype RAW264.7 with CXCL1 overexpression, followed by XIAOPI formula treatment). Throughout the treatment, mice were weighed and their tumors were measured with a caliper every 3 days. Tumor volumes (V) were calculated using the formula: V = (length) × (width)^2^/2. D-luciferin (PerkinElmer, Boston, USA) at 150 mg/kg was injected intraperitoneally for luminescent imaging. Mice were imaged using the IVIS-Spectrum system (PerkinElmer, Boston, USA) every week to monitor lung metastasis. After 6 weeks, the mice were euthanized and the tumors and lungs were removed. Primary cells were isolated from fresh tumors and lungs and subjected for TAMs analysis as indicated above by flow cytometry. In addition, from the second week of inoculation onwards, the recruitment of HSPC in the bone marrow of mice was detected weekly by flow cytometry. Hematoxylin and Eosin staining was performed to detect micro-metastasis in lung tissue. The MDSCs population in tumors and lungs was also monitored by flow cytometry as indicated above.

### Hematoxylin and eosin staining

Tumor specimens were fixed in 4% paraformaldehyde for 24 h, followed by the protocol as we described previously [17]. Hematoxylin and Eosin staining was conducted using the Hematoxylin and Eosin Staining Kit (Beyotime Biotechnology, Shanghai, China) according to the manufacturer’s instructions.

### Statistical analysis

Data were presented as mean ± standard deviation (SD). All statistical analyses were performed using Statistical Product and Service Solutions (SPSS) 20.0 software (Abbott Laboratories, Chicago, USA). The one-way ANOVA and the Dunnett’s post hoc test were performed for comparison among multiple groups. ANOVA for repeated measurement was performed towards repeated measures data. *P* < 0.05 was considered as statistically significant.

## Results

### XIAOPI formula inhibits the polarization of M2 macrophages and CXCL1 expression

**Fig. 1:**
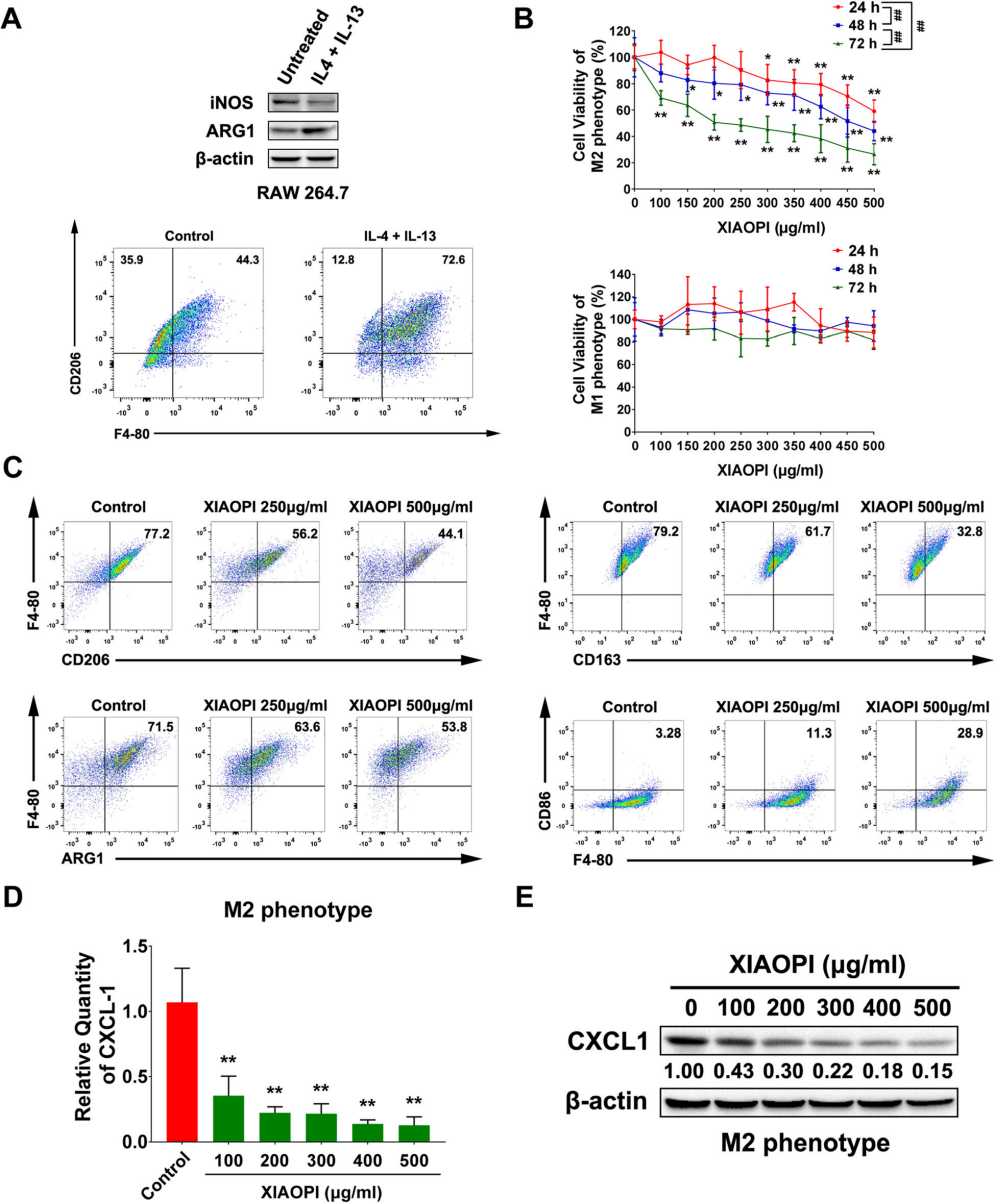
XIAOPI formula suppresses the polarization of M2 macrophages and CXCL1 expression. **a** The phenotype changes of RAW264.7 induced by 40 ng/ml IL-4 and 40 ng/ml IL-13. **b** Effects of XIAOPI formula on the proliferation of M1 and M2 phenotype RAW264.7. **c** The surface marker CD206, CD163 and Arg1 of M2 phenotype were all downregulated dose-dependently under the treatment of XIAOPI formula, while the M1 marker CD86 was upregulated. **d** XIAOPI formula dose-dependently downregulated CXCL1 transcription in M2 phenotype RAW264.7 cells. **e** XIAOPI formula dose-dependently inhibited expression of CXCL1 in M2 phenotype RAW264.7 cells. (The results were obtained from triplicate experiments and were represented as mean values ± SD., *P < 0.05, **P < 0.01 as compared with control, ^##^*P* < 0.01, comparison between three time points of 24 h, 48 h, and 72 h)

Macrophages could be developed into M2 phenotype (usually termed as tumor-associated macrophages, TAMs) induced by cytokines such as IL-4 and TGF-β. Therefore, we firstly applied IL-4 and IL-13 to stimulate RAW264.7 into M2 phenotype in vitro. As verified, the level of M1 marker iNOS was decreased, while the expression of M2 marker ARG1 was enhanced. Meanwhile, it was also observed that the proportion of M2 antigen CD206 was elevated by flow cytometry analysis (Fig. 1a). Our previous study suggested that XIAOPI formula could suppress breast cancer growth and lung metastasis via interrupting TAMs/CXCL1 signaling [16]. We, therefore, need to confirm the effects of XIAOPI formula on the viability, polarization and CXCL1 expression on TAMs. It was found that the proliferation of M2 phenotype RAW264.7 macrophages was suppressed by XIAOPI formula in a time- and dose-dependent manner. However, different from the M2 macrophages, XIAOPI formula had no obvious inhibitory effect on the proliferation of M1 macrophages, which implied that XIAOPI might be selective for M2 macrophages, not a generalized immunotoxicity (Fig. 1b). On the other hand, the expression of CD163, CD206, and Arg1 of M2 macrophages were downregulated gradually with the increasing dosage of XIAOPI formula, suggesting that XIAOPI formula blocked the polarization of M2 macrophages (Fig. 1c). In addition, XIAOPI administration also resulted in an increased CD86 expression, indicating that XIAOPI formula might also promote the repolarization of M2 macrophages (Fig. 1c). Since CXCL1 was demonstrated as the highest chemokine secreted by TAMs, we also found that XIAOPI formula dose-dependently restrained the transcription and expression of CXCL1 of M2 phenotype macrophages as well (Fig. 1d & e). All these findings demonstrated that XIAOPI formula could effectively inhibit TAMs/CXCL1 signaling in vitro.

### XIAOPI formula inhibits HSPCs mobilization and MDSCs differentiation via inhibiting TAMs/CXCL1

**Fig. 2:**
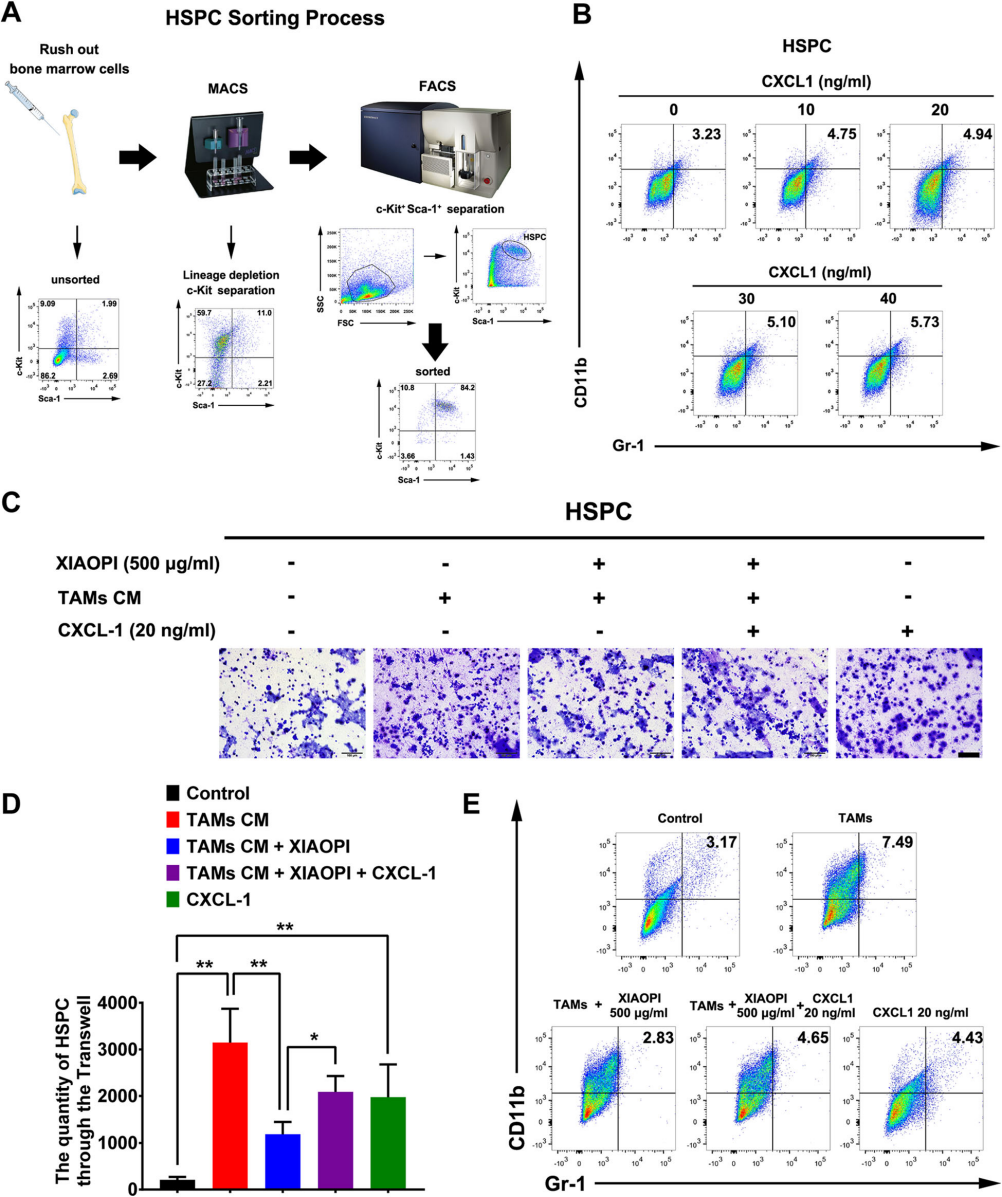
XIAOPI formula inhibits HSPCs recruitment and MDSCs differentiation via inhibiting TAMs/CXCL1. **a** Flow chart of HSPCs preparation process. Briefly, murine bone marrow cells were flushed out by a syringe under sterile conditions. Lineage^−^/c-Kit^+^ magnetic cell sorting was subsequently conducted. Finally, Sca-1^+^/c-Kit^+^ HSPCs were collected using FACS. **b** CXCL1 dose-dependently enhanced the proportion of CD11b^+^/Gr-1^+^ cells, facilitated the differentiation of HSPCs towards MDSCs. **c-d** XIAOPI formula significantly attenuated the movement of HSPCs induced by TAMs-CM, which was relieved by CXCL1 administration. **e** XIAOPI formula restrained the differentiation of HSPCs to CD11b^+^/Gr-1^+^ MDSCs induced by TAMs-CM and was also reduced by CXCL1 treatment. (The results were obtained from triplicate experiments and were represented as mean values ± SD, *P < 0.05, **P < 0.01)

HSPCs were reported to play a critical role in mediating the formation of PMN. Herein, we used MACS combined with FACS strategies to separate HSPCs labeled with Lineage^−^/c-Kit^+^/Sca-1^+^ from the bone marrow of mice (Fig. 2a). Since HSPCs recruitment and mobilization depended on chemokines secreted by tumor or suppressive immune cells and CXCL1 was validated to promote distant metastasis [11, 18], we, therefore, detected the effects of CXCL1 on the differentiation of HSPCs. Flow cytometry results indicated that CXCL1 administration gradually increased the population of CD11b^+^/Gr-1^+^ MDSCs in HSPCs (Fig. 2b). Furthermore, it was revealed that the conditional medium (CM) of TAMs could increase the invasion ability of HSPCs, indicating that TAMs could activate the directional recruitment of HSPCs. By contrast, XIAOPI formula significantly blocked the invasion enhancement of HSPCs induced by TAMs, and CXCL1 further relieved this suppression, implying that CXCL1 is a critical chemokine secreted by TAMs accounting for HSPCs recruitment (Fig. 2c&d). In addition, TAMs-CM was also found to promote HSPCs differentiation to MDSCs and was blocked by XIAOPI formula administration. Similarly, CXCL1 treatment declined the suppression effects of XIAOPI formula on MDSCs differentiation (Fig. 2e). Since MDSCs were considered as a necessary contributor of PMN formation, these findings suggested that XIAOPI formula might be effective in suppressing PMN formation via inhibiting HSPCs recruitment and MDSCs differentiation.

### XIAOPI formula inhibits proliferation and invasion of 4 T1 cells co-cultured with TAMs and HSPCs

**Fig. 3:**
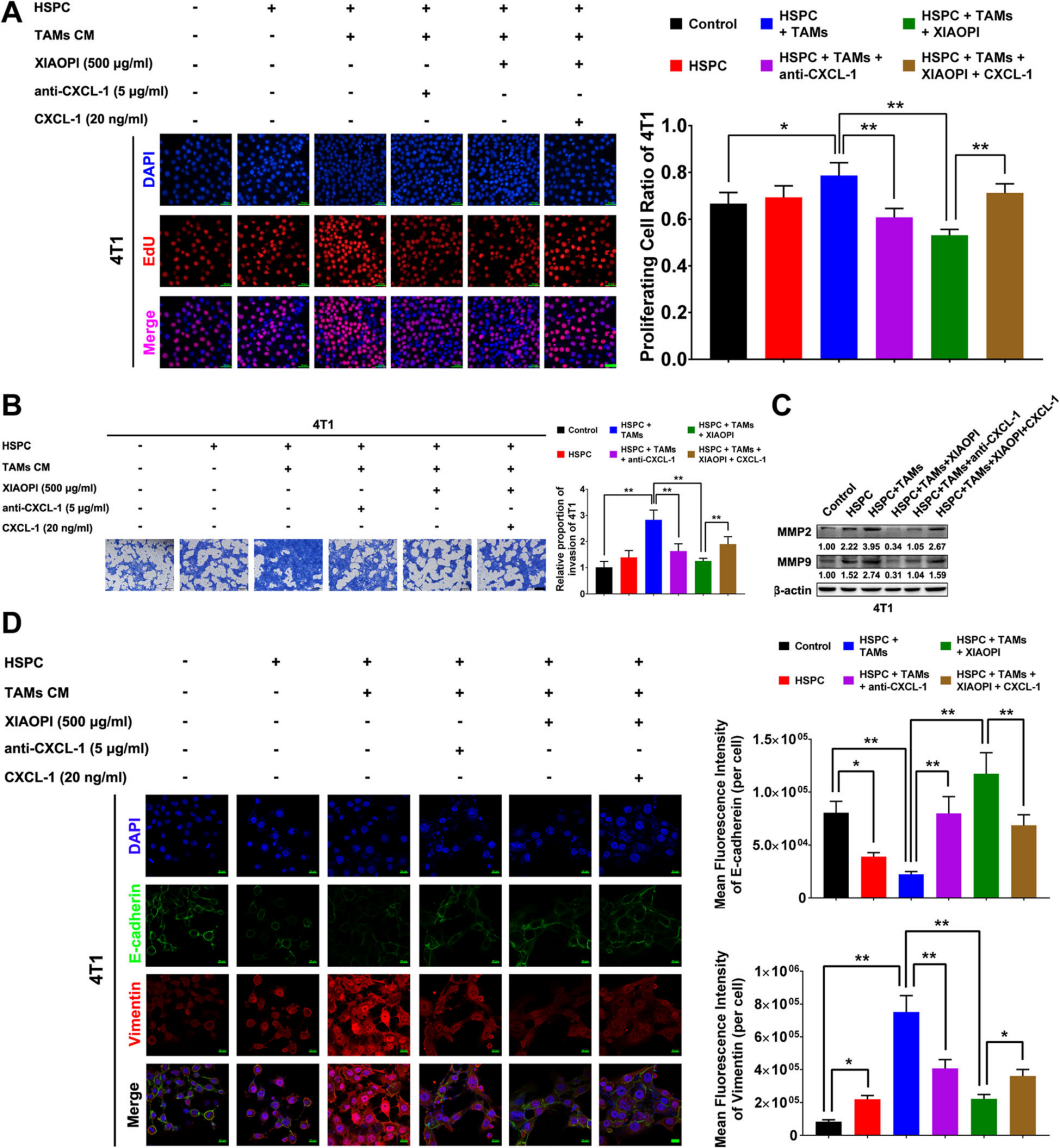
XIAOPI formula inhibits the proliferation and invasion of 4 T1 cells co-cultured with TAMs and HSPCs. **a** EdU cell proliferation assay demonstrated the co-culture of HSPCs and TAMs-CM promoted the proliferation of 4 T1, while was inhibited by XIAOPI formula or CXCL1 neutralizing antibody (the scale bars indicate 50 μm). **b** The invasion of 4 T1 cells was significantly promoted by co-culture of HSPCs and TAMs-CM, while was inhibited by XIAOPI formula treatment and CXCL1 neutralizing antibody. Meanwhile, the suppressive effects of XIAOPI was reversed by exogenous CXCL1 treatment (the scale bars indicate 200 μm). **c** Co-culture with HSPCs and TAMs-CM significantly promoted the expression of MMP2 and MMP9, while XIAOPI formula or CXCL1 neutralizing antibody reduced the elevated levels of MMP2 and MMP9. However, exogenous CXCL1 could partly restore MMP2 and MMP9 levels that were suppressed by XIAOPI formula. **d** Quantification of fluorescence intensity in 4 T1 cells (per cell) indicated that XIAOPI formula and CXCL1 neutralizing antibody inhibited the mesenchymal marker vimentin, which was upregulated by co-culture with HSPCs and TAMs-CM. By contrast, the expression of epithelial marker E-cadherin was enhanced (the scale bars indicate 20 μm). (The results were obtained from triplicate experiments and were represented as mean values ± SD, *P < 0.05, **P < 0.01)

The above results indicated that the XIAOPI formula could block the formation of tumor immune suppression microenvironment via inhibiting MDSCs. It is interesting to explore the formula’s influences on cancer cell proliferation and metastasis under the condition simulating the tumor microenvironment (TME). HSPCs and TAMs-CM were added to the supernatants of mouse cancer cell line 4 T1 either alone or in combination. EdU cell proliferation assay indicated that HSPCs alone had little influence on 4 T1 proliferation, but TAMs-CM-treated HSPCs could significantly enhance that. When CXCL1 neutralizing antibody was added to the co-culture system, the proliferation promoting effects were inhibited, indicating CXCL1 is a critical chemokine in TAMs-CM activating HSPCs-mediated cancer cell proliferation. Notably, the proliferation-promotion effects of TAMs-CM-treated HSPCs on cancer cells were significantly inhibited following XIAOPI administration, and were subsequently reversed by CXCL1 overexpression (Fig. 3a). These findings further demonstrated that TAMs/CXCL1 signaling is a critical chemokine regulating HSPCs function and the anti-cancer effects of XIAOPI formula. Transwell assay also revealed that TAMs-CM-treated HSPCs enhanced the invasion ability of 4 T1 cells, and was suppressed by XIAOPI treatment or CXCL1 neutralizing. Similarly, the inhibitory effects of XIAOPI formula were relieved by CXCL1 overexpression (Fig. 3b). In addition, XIAOPI formula also inhibited the upregulation of MMP2 and MMP9 expression induced by TAMs-CM-treated HSPCs, thus impaired the extracellular matrix remodeling during the formation of premetastatic niches. Similarly, the exogenous CXCL1 could partly restore MMP2 and MMP9 levels that were suppressed by XIAOPI formula, suggesting that XIAOPI might inhibit the invasion of breast cancer cells in a CXCL1 dependent manner. (Fig. 3c). Immunofluorescence experiments further indicated that TAMs-CM-treated HSPCs promoted the expression of mesenchymal marker vimentin and downregulated the level of epithelial marker E-cadherin. On the contrary, XIAOPI formula suppressed the EMT process by inhibiting CXCL1 (Fig. 3d). These results implied that XIAOPI formula could inhibit the proliferation and metastatic ability of cancer cells in the TME, and therefore resulting in the suppression of PMN formation.

### XIAOPI formula inhibits breast cancer lung metastasis

**Fig. 4:**
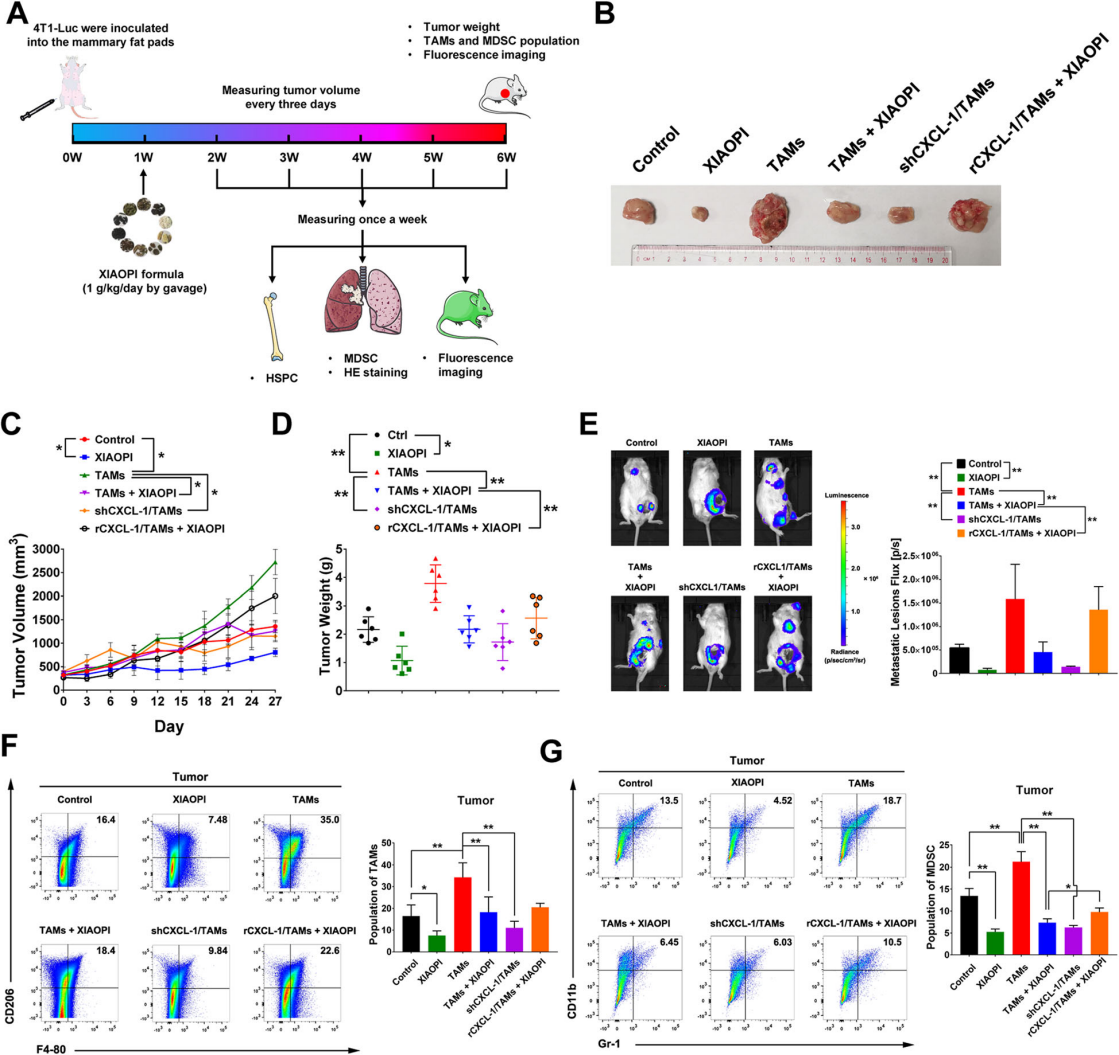
XIAOPI formula inhibits breast cancer lung metastasis and reduces the proportions of MDSCs and TAMs in the tumor. **a** Schematic diagram of animal model establishment, drug administration and parameters tested. **b** Representative image of breast tumors under different treatments. **c-d** XIAOPI formula inhibited breast cancer growth and suppressed the growth promotion effects induced by TAMs coinjection. Meanwhile, CXCL1 knockdown in TAMs was found to restrain the growth-stimulating effects of TAMs on breast cancer, while CXCL1 overexpression in TAMs was found to relieve the inhibitory effects of XIAOPI formula. **e** XIAOPI formula inhibited breast cancer lung metastasis and suppressed the promotion effects induced by TAMs coinjection. Meanwhile, CXCL1 knockdown in TAMs was found to limit the metastasis-stimulating effects of TAMs on breast cancer, while CXCL1 overexpression in TAMs was found to block the inhibitory effects of XIAOPI formula. **f** XIAOPI formula could inhibit the M2 type macrophages in the primary breast tumors or TAMs-stimulated breast tumors, CXCL1 knockdown in TAMs could downregulate the population of M2 phenotype populations and CXCL1 overexpression in TAMs relieved the suppression effects of XIAOPI formula. **g** XIAOPI formula could inhibit the MDSCs induced by 4 T1 cells or TAMs stimulation, CXCL1 knockdown in TAMs could downregulate the population of MDSCs and CXCL1 overexpression in TAMs relieved the suppression effects of XIAOPI formula. (The results were represented as mean values ± SD, *P < 0.05, **P < 0.01)

In order to confirm the in vitro results, we further evaluated anti-metastasis effects of XIAOPI formula in vivo by injecting luciferase-labeled 4 T1 cells into the mammary fat pads of female BALB/c mouse. XIAOPI formula was given by oral gavage at 1 g/kg/day from the first week to the ending of experiments. The lung metastasis of breast cancer was monitored using fluorescence imaging every week, and the formation of micro-metastases in the lung was examined by Hematoxylin and Eosin staining. Moreover, the proportion of HSPCs from murine bone marrow and MDSCs in tumors and lung tissues were detected (Fig. 4a). The volume of the tumor was measured every 3 days. It was found that XIAOPI formula significantly inhibited the orthotropic cancer growth, and could suppress the cancer promotion effects induced TAMs. What’s more important, CXCL1 overexpression in TAMs significantly reduced the suppression effects of XIAOPI formula (Fig. 4b-d). Luciferase-imaging experiments further revealed that XIAOPI formula or CXCL1 knockdown could significantly block TAMs-induced cancer cells metastasis, and the anti-metastasis effects of XIAOPI formula were abrogated by CXCL1 overexpression in TAMs (Fig. 4e). These results further demonstrated that TAMs/CXCL1 is a key target of XIAOPI formula to inhibit breast cancer metastasis. Since TAMs were considered as the most enriched cell component in tumor stroma, we therefore detected the effects of XIAOPI on its proportion in tumors by flow cytometry. The results showed that XIAOPI formula and CXCL1 knockdown in TAMs could significantly inhibit F4–80^+^ CD206^+^ populations in tumors, even in the group of TAMs co-injection with 4 T1 cells. Similarly, co-injection of CXCL1-overexpressing TAMs blocked the inhibitory effects of XIAOPI formula on TAMs population (Fig. 4f). On the other hand, the MDSCs population in tumor tissues was also inhibited by XIAOPI treatment or CXCL1 knockdown in TAMs. Importantly, XIAOPI formula could inhibit the increased MDSCs population induced by TAMs, which was subsequently reversed by CXCL1 overexpression (Fig. 4g). These results suggested that TAMs/CXCL1 plays a critical role in mediating the anti-cancer and metastasis inhibition effects of XIAOPI formula, which was closely associated with downregulation of TAMs and MDSCs in tumor tissues.

### XIAOPI formula inhibits the formation of TAMs-induced PMN in mice by suppressing CXCL1-mediated MDSCs activation

**Fig. 5:**
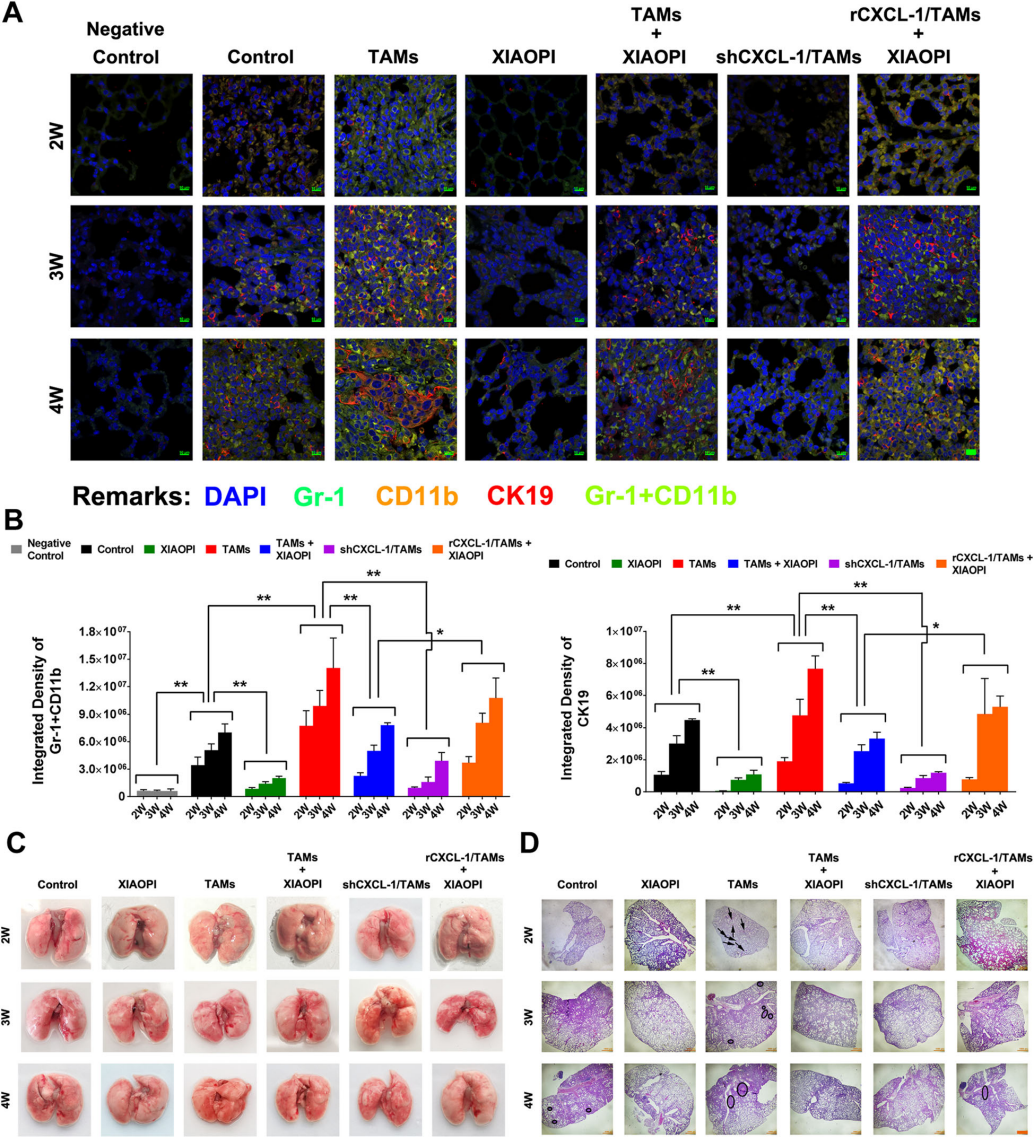
XIAOPI formula inhibits TAMs-induced PMN formation in mice via suppressing CXCL1-mediated MDSCs activation. **a** Immunofluorescence of lung tissues of mice from the 2nd to 4th weeks after inoculation. Gr-1^+^ CD11b^+^ labeled MDSCs and CK19^+^ labeled cancer cells. XIAOPI formula treatment remarkably inhibited CD11b^+^/Gr1^+^ MDSCs accumulation in the lung tissues, but was relieved by CXCL1 overexpression in TAMs. **b** Statistics of the fluorescence intensity of Gr-1, CD11b, and CK19 in lung tissue of mice from the 2nd to 4th weeks. **c** Gross observation of mouse lung tissue from the 2nd to 4th weeks. There were no apparent lung metastatic lesions were noticed. **d** Hematoxylin and Eosin staining of mouse lung tissue from the 2nd to 4th weeks. XIAOPI formula inhibited TAMs-induced micro-metastatic sites in the lung. (The results were represented as mean values ± SD, *P < 0.05, **P < 0.01)

PMN was considered as a necessary condition facilitating the formation of metastatic lesions. In order to determine the effects of XIAOPI formula on PMN formation, Gr-1 and CD11b were used to label MDSCs and CK19 was applied to mark cancer cells. Immunofluorescence results found that TAMs co-injection began to induce the CD11b^+^Gr1^+^ MDSCs accumulation as early as the 2nd week, accompanied by the infiltration of CK19^+^ cancer cells in the lung PMN. However, the TAMs-induced MDSCs accumulation and cancer cell infiltration was significantly blocked following XIAOPI treatment or CXCL1 knockdown. On the contrary, CXCL1 overexpression in TAMs reversed the PMN inhibitory effects of XIAOPI formula (Fig. 5a). From the 2nd to 4th weeks of tumor progression, the more CD11b^+^ Gr1^+^ MDSCs accumulated in the lung tissue, the more CK19^+^ breast cancer cells infiltrated, indicating that PMN formation provides a positive condition for cancer cell seeding. The statistical differences between groups remain consistent from the 2nd to 4th week (Fig. 5b). In addition, we noticed that there were no apparent lung metastatic lesions from the 2nd to 4th weeks in all groups as showed in Fig. 5c, but HE staining revealed that XIAOPI formula could inhibit the TAMs-induced formation of micro-metastatic niches in the lung, further supporting that XIAOPI formula could prevent metastasis by inhibiting PMN formation (Fig. 5d).

### XIAOPI formula inhibits HSPCs recruitment and differentiation into MDSCs

**Fig. 6:**
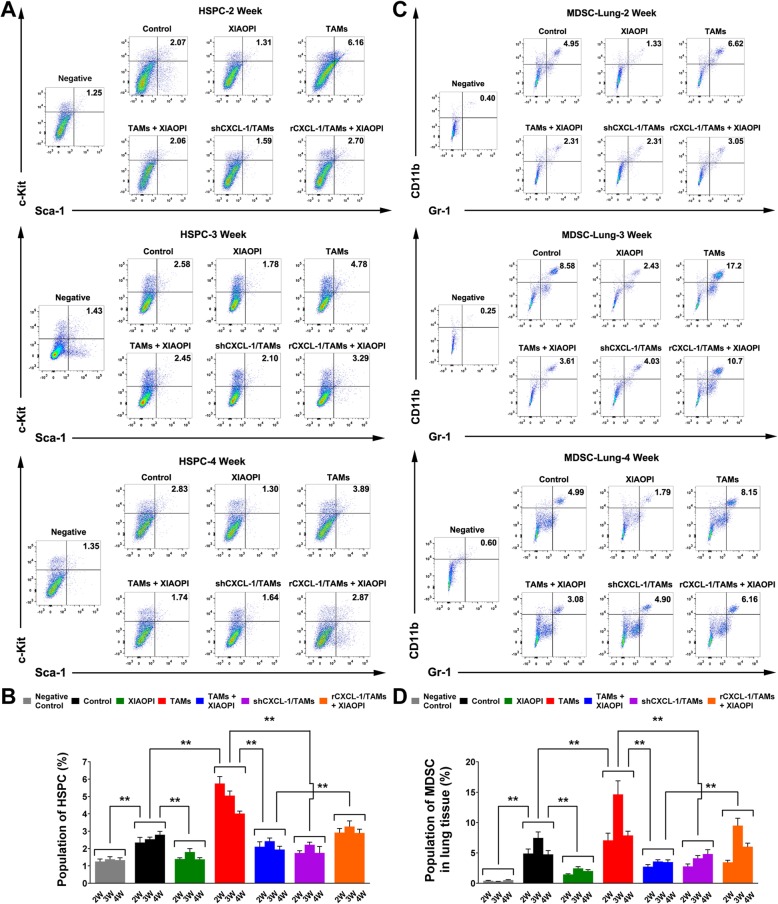
XIAOPI formula inhibits the recruitment of HSPCs and differentiation into MDSCs. **a-b** The HSPCs populations in the bone marrow were detected by flow cytometry. XIAOPI formula treatment blocked TAMs-induced HSPCs elevation, but was relieved by CXCL1 overexpression. **c-d** The MDSCs populations in the lung tissues were detected by flow cytometry. XIAOPI formula treatment remarkably inhibited CD11b^+^/Gr1^+^ MDSCs accumulation in the lung tissues from the 2nd to 4th weeks, but was relieved by CXCL1 overexpression in TAMs. (The results were represented as mean values ± SD, *P < 0.05, **P < 0.01)

The above findings indicated that XIAOPI formula could inhibit MDSCs activation and accumulation in target organs, since MDSCs were reported to differentiate from HSPCs, it is interesting to explore the changes of HSPCs responding to TAMs inoculation or XIAOPI treatment. As showed in Fig. 6a & b, the population of HSPCs was significantly elevated from the 2nd to 4th weeks following cancer cells inoculation compared to the negative control group. What’s more important, HSPCs was further activated by TAMs co-injection, and CXCL1 knockdown could inhibit the process. Intriguingly, XIAOPI treatment could remarkably suppress the cancer cell-induced or TAMs-induced HSPCs enrichment throughout the in vivo experiment, and CXCL1 overexpression declined the inhibitory effects of XIAOPI formula on HSPC activation, further implying that TAMs/CXCL1 is a critical signaling accounting for the anti-PMN effects of XIAOPI. Corresponding to HSPCs changes in the bone marrow, flow cytometry analysis revealed that the MDSCs population in the lung tissues remained the similar change tendency with HSPCs from the 2nd to 4th weeks. The MDSCs population was also triggered by TAMs inoculation and downregulated by CXCL1 knockdown. Meanwhile, XIAOPI treatment could inhibit the elevated MDSCs induced by TAMs, and CXCL1 overexpression blocked the XIAOPI formula’s therapeutic efficacy (Fig. 6c & d). Therefore, XIAOPI formula was demonstrated to inhibit the recruitment and differentiation of HSPCs, thus subsequently suppress the PMN formation to prevent breast cancer metastasis.

## Discussion

Recent studies suggested that PMN formation is a critical factor determining the site and time of cancer metastasis [19, 20]. Drugs targeting PMN could represent a novel therapeutic strategy in improving clinical outcomes of cancer patients. However, PMN formation is a complex procedure that involves multiple cytokines, bone marrow cells and tissue remodeling in premetastatic organs [21]. Traditional cell killing strategies or targeted therapy might be not enough to block the network reactions. TCM, which contains multiple ingredients that can simultaneously act on multiple targets, has long been tested for cancer adjuvant prevention and treatment due to its systematic regulation on immune function [22–26]. Multiple studies have demonstrated that conventional formulas could inhibit cancer drug resistance or metastasis via regulating T cell phenotype, macrophage polarization, chemokine expression and immune checkpoint [27, 28]. Meta-analyses of clinical trials have also demonstrated that traditional medical practice could improve cancer patients’ survival period and life quality, accompanied by normalization of tumor immune microenvironment [14, 29–32]. Meanwhile, a number of TCM formulas have been commercialized and prescribed to cancer patients with positive clinical efficacy, such as Huaier granule, PHY906 and Huachansu, which shows great effects on tumor microenvironment modulation including macrophage inhibition, T cell differentiation and cytokine secretion [30, 33, 34]. Our study demonstrated that XIAOPI formula could inhibit breast cancer PMN formation by inhibiting TAMs/CXCL1 signaling.

TAMs are usually characterized into classically activated M1 phenotype macrophage and alternatively activated M2 phenotype macrophage [35]. Generally, M1 was identified as “Inhibit” type due to their function to promote Th1 responses with microbicidal and tumoricidal effects, while M2 was considered as “Heal” type given their effects to activate Th2 responses and promote tissue repair and remodeling, angiogenesis and immune suppression, as well as tumor progression [36]. Therefore, inhibition M2 phenotype polarization has been considered as an important strategy for cancer treatment. In the present study, we found that TAMs could facilitate breast cancer growth and metastasis in vitro and in vivo. The CM of TAMs could induce HSPCs recruitment and differentiation into MDSCs, which further supported PMN formation. In 2015, Kaplan et al tracked the developmental fate of HSPCs in tumor-bearing mice. It was found that distant primary tumors drove the expansion of HSPCs in the bone marrow and their mobilization into the bloodstream. Further tracing of the purified HSPCs in vivo showed that they differentiated into MDSCs at the early metastatic site of tumor-bearing mice [12]. In addition, another study by Kaplan et al suggested that VEGFR1-positive hematopoietic progenitor cells are necessary in the regulation of tumor metastasis [3]. CXCL1 has also been shown to indirectly mobilize HSPCs into the peripheral blood from bone marrow via MMP9 release from neutrophils [37, 38]. Our study showed that CXCL1 could promote the recruitment of HSPCs and its differentiation into MDSCs. However, further studies are needed to investigate the precise mechanisms by which CXCL1 mobilizes and expands HSPCs.

Of course, CXCL1 is not the only cytokine that induces HSPCs to differentiate into MDSCs [12]. Previous findings suggested that multiple factors secreted by TAMs, such as TGF-β, SDF-1 and VEGF, could facilitate PMN formation in the distant organ [39]. In particular, GM-CSF and IL-6 could not only promote the myeloid-biased differentiation, but also induce the differentiation of myeloid precursors into functional MDSCs [40]. However, our previous findings demonstrated that CXCL1 acts as the highest chemokine secreted by TAMs isolated from breast cancer [16, 41]. Moreover, CXCL1 silencing in TAMs was found to inhibit breast cancer growth and lung metastasis via inhibiting NF-κB/SOX4 signaling in both murine and human models [42]. Similar results were also observed in other malignancies. CXCL1 derived from TAMs and cancer associated fibroblasts could promote the invasion and colonization of bladder cancer cells [43]. In addition, VEGF-A secreted by colon cancer cells could stimulate TAMs to produce CXCL1, which subsequently recruited MDSCs into the premetastatic sites to establish PMN, and in turn promoting liver metastasis [11]. It was also reported that CXCL1 could attract CD11b^+^ Gr1^+^ myeloid cells into the tumor, resulting in the production of chemokines including S100A8/9 that enhancing tumor growth and metastasis [21]. Clinically, the tissue microarray analysis of 276 patients with colon cancer indicated that CXCL1 played a vital role in the progression and metastasis of colon cancer [44]. The expression level of CXCL1 is an independent prognostic factor for both OS and DFS in patients with colon cancer [45]. Therefore, disrupting the TAMs/CXCL1 signaling to block PMN formation is emerging as a promising therapeutic approach for cancer metastasis. Nevertheless, cocktail therapy targeting multiple cytokines is still worth to develop in the future.

Activation of bone marrow-derived cells is considered as a central step in establishing PMN. Previous studies have shown that HSPCs recruitment and MDSCs differentiation were important prerequisites mediating PMN formation [12]. In our study, it was also observed that HSPCs in the bone marrow were significantly recruited after 2 weeks of 4 T1 cells inoculation in mice, while the MDSCs in the lungs were also upregulated. The findings suggested that PMN was established in the early stage of breast cancer. Interestingly, it was noticed that HSPCs population was not always being elevated. Six weeks after inoculation of 4 T1 cells, HSPCs were found to appear a sharp decrease. This was consistent with the findings of Liu et al. that HSPCs population was gradually decreased after the formation of tumor colonies in target organs of metastasis [19]. Moreover, TAMs co-inoculation was found to promote HSPCs recruitment and MDSCs differentiation, which was blocked by XIAOPI formula and CXCL1 knockdown. These findings suggested that TAMs/CXCL1 signaling is a crucial messenger triggering bone marrow-derived cells. Interestingly, HSPCs population in XIAOPI-treated group was maintained stable throughout the whole experiment. The results not only indicated that XIAOPI could inhibit HSPCs elevation in the early stage of breast cancer, but also bring little hematopoietic toxicity effects during treatment.

TCM is particularly appreciated for cancer adjuvant therapy in East Asia. Different from traditional chemotherapy drugs and targeted inhibitors, TCM is characterized by holistic regulation and network intervenes [22, 23]. Numerous studies demonstrated that TCM formula could not only synergistically enhance the therapeutic efficacy of cytotoxic drugs, but also reduce their side effects [24–26]. Moreover, multiple clinical studies approved that the survival period of TCM users is greatly prolonged compared to TCM non-users, which is closely correlated with immunity improvement. For example, KSG-002, a new herb formula, was reported to inhibit breast cancer growth and metastasis by downregulating NF-κB-dependent TNFα secretion derived from TAMs [46]. Huaier extract was also found to inhibit M2 macrophages infiltration and angiogenesis [47]. These findings suggested that TME is a central regulatory target of TCM. However, studies focusing on the PMN inhibitory effects of TCM were rarely reported. It is noteworthy that a study investigated the regulation effects of the Jianpi Bushen formula on the expression changes of PMN biomarkers in gastric cancer. It was found that the PMN signals including Rac1, Cdc42, SDF-1 and FN were all inhibited by Jianpi Bushen [48], but the study was too descriptive and did not detect the population changes of HSPCs and MDSCs. Our study found that XIAOPI formula could dose-dependently inhibit M2 polarization and CXCL1 expression. Moreover, XIAOPI was capable of inhibiting TAMs/CXCL1-induced HSPCs recruitment and MDSCs differentiation, and finally limiting the PMN formation and lung metastasis. Our study further provided scientific evidence supporting the application of TCM in preventing breast cancer metastasis. Besides, there are no specific commercial CXCL1 inhibitors at present, and current targeting strategy was designed by acting on its receptor CXCR2, implying that the inhibition of CXCL1 is not specific. However, our study demonstrated that XIAOPI formula could inhibit CXCL1 mRNA and protein expression. More importantly, our previous studies indicated that XIAOPI formula suppressed the promoter activity of CXCL1 gene in TAMs [41]. Meanwhile, XIAOPI formula contains thousands of phytochemicals and molecular targets, which shows a natural advantage than CXCL1 mono-targeting strategy. In addition, XIAOPI formula showed little adverse events in our study and already approved by China State Food and Drug Administration, but it was remained unclear what kind of compound in XIAOPI formula accounting for CXCL1 inhibition. It is still worth to discover and identify CXCL1 inhibitor from XIAOPI formula or natural chemical libraries in the future.

## Conclusion

Taken together, our study demonstrated that XIAOPI formula could prevent breast cancer PMN formation and lung metastasis via inhibiting TAMs/CXCL1 signaling. Our findings not only provided preclinical evidence supporting XIAOPI application in preventing PMN formation, but also highlighted the novel role of TAMs/CXCL1 in mediating PMN establishment.

## Data Availability

The datasets used and/or analyzed during the current study are available from the corresponding author on reasonable request.

## Abbreviations

BMDCs: Bone marrow-derived cells
CM: Conditional medium
FBS: Fetal bovine serum
HSPCs: Hematopoietic stem/progenitor cells
MDSCs: Myeloid-derived suppressor cells
PMN: Premetastatic niche
TAMs: Tumor associated macrophages
TCM: Traditional Chinese medicine
TME: Tumor microenvironment
